# Meckel's diverticulum band adhesion causing internal hernia, small bowel obstruction complicated by peritonitis and massive ascites: a rare pediatric case

**DOI:** 10.3389/fped.2026.1834207

**Published:** 2026-07-20

**Authors:** Jinli Su, Hailong Zhang

**Affiliations:** Maternal and Child Health Hospital of Inner Mongolia Autonomous Region, Inner Mongolia Autonomous Region, Hohhot, China

**Keywords:** case report, children, extremely rare, gastrointestinal tract, Meckel's diverticulum

## Abstract

Meckel's diverticulum (MD) is one of the most common congenital anomalies of the gastrointestinal tract in children, although it may occasionally be detected in adults. It is typically located within 100 cm of the ileocecal valve in the distal ileum. The diverticulum varies in size and morphology; many patients remain asymptomatic throughout life. In children, the most common clinical presentation is painless gastrointestinal bleeding, followed by intestinal obstruction. Intussusception may also occur. Once complications develop, patients often present with acute abdomen. Failure to establish a timely diagnosis and initiate appropriate management may result in intestinal ischemia or even necrosis. Furthermore, acute intestinal obstruction may obscure the underlying pathology, increasing the risk of misdiagnosis or missed diagnosis on radiologic evaluation. We report a rare case of Meckel's diverticulum complicated by band adhesion resulting in internal hernia and small bowel obstruction, further associated with peritonitis and massive ascites. The diverticulum was located approximately 40 cm proximal to the ileocecal valve. A fibrous diverticular band adhered to adjacent bowel loops, forming a hernia orifice that caused obstruction of the neighboring small intestine and mesentery. The patient underwent surgical resection of the Meckel's diverticulum, partial ileal resection, and end-to-end ileo-ileal anastomosis. The procedure was uneventful, and postoperative recovery was satisfactory. Internal hernia caused by Meckel's diverticulum band adhesion with associated peritonitis and massive ascites is rare. This case broadens the differential diagnosis of pediatric intestinal obstruction without prior abdominal surgery.

## 1 Introduction

Acute intestinal obstruction in children without prior abdominal surgery is a common surgical emergency. Imaging plays a critical role in evaluation and diagnosis (1–3). Meckel's diverticulum (MD) is a relatively uncommon etiology and is often discovered only when complications such as obstruction, diverticulitis, perforation, or umbilical anomalies occur (4, 5), thereby masking the true underlying pathology (6).

Plain abdominal radiography is typically the initial modality for assessing intestinal obstruction and possible perforation. Computed tomography (CT) provides a more comprehensive evaluation of abdominopelvic anatomy (7), particularly in diagnosing complex causes such as internal hernia.

Meckel's diverticulum, also known as distal ileal diverticulum, results from incomplete obliteration of the omphalomesenteric (vitelline) duct during the fifth week of gestation (8). A Meckel's diverticulum band (MDB) represents an embryologic remnant of the vitelline vessels. It typically extends from the root of the mesentery or between the mesentery and terminal ileum to the apex of the diverticulum or distal ileum, forming a fibrous band adhesion that can cause intestinal obstruction (9). Zhang et al. (10) reported that MDB appears as fibrous cord-like structures of varying length and thickness, measuring approximately 3–7 mm in diameter, and may attach to the mid-mesentery, terminal ileal mesentery, or terminal ileum. An ileal loop may herniate through the opening formed between the band and the mesentery, resulting in bowel compromise. Prolonged strangulation may lead to ischemic necrosis.

## 2 Case report

### 2.1 Patient information and clinical findings

A 6-year-old girl presented to the emergency department with persistent acute abdominal pain for 20 h. She had no history of abdominal surgery. Prior evaluation at a local hospital with plain radiography suggested intestinal obstruction, and she was transferred to our institution.

### 2.2 Diagnostic assessment

**Figure 1:**
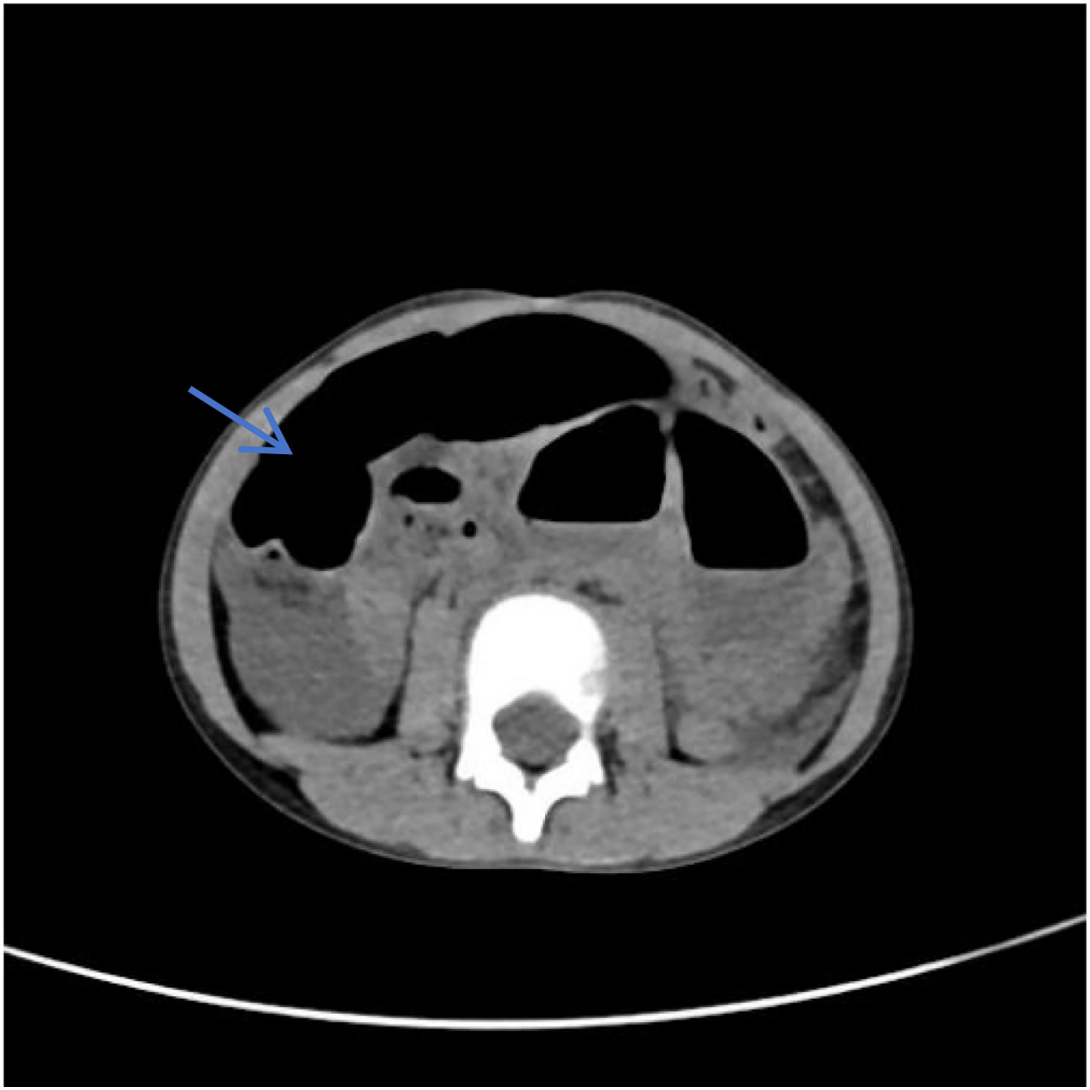
Small bowel obstruction with multiple air-fluid levels.

**Figure 2:**
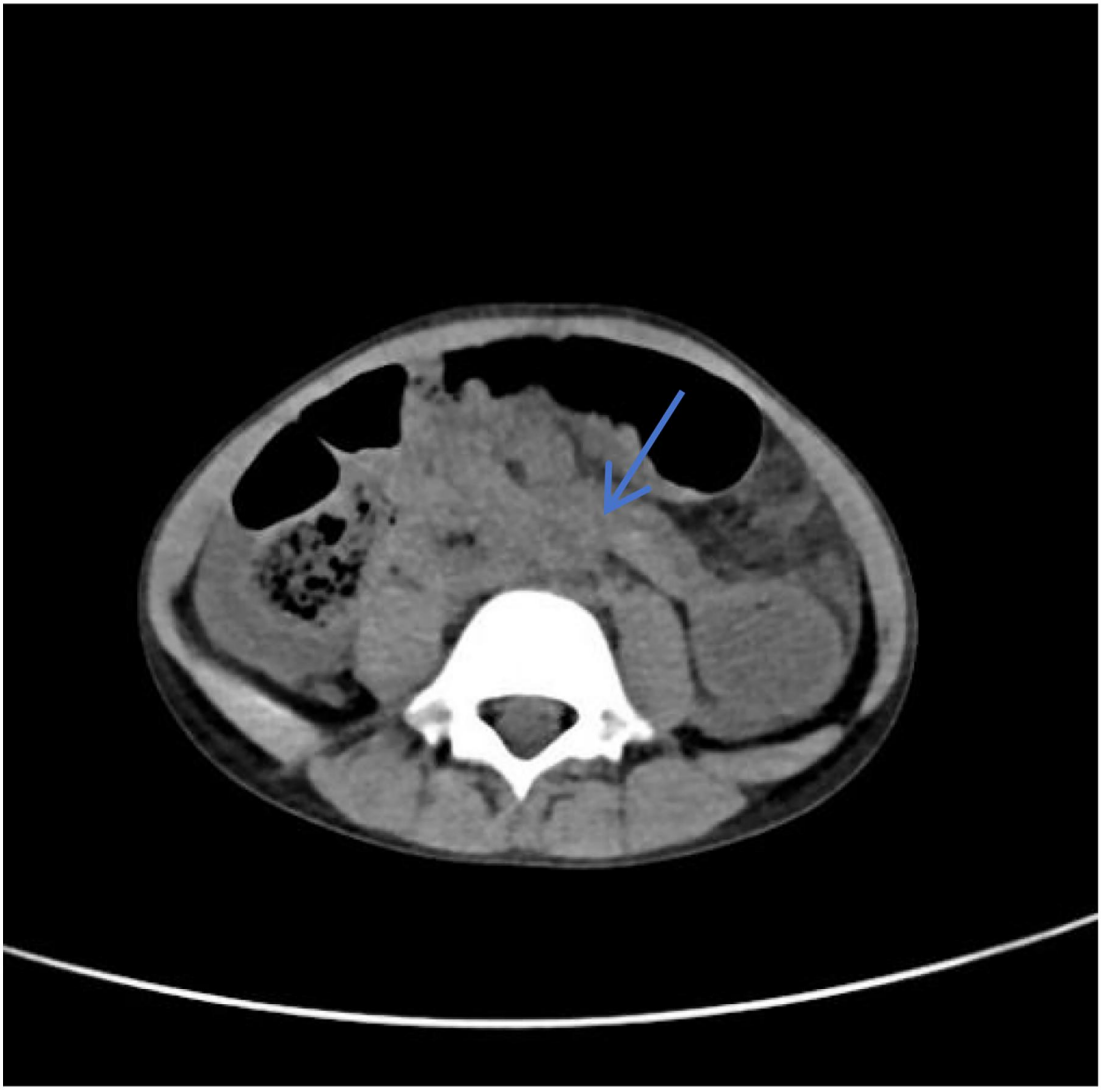
Internal hernia with whirl sign formation.

**Figure 3:**
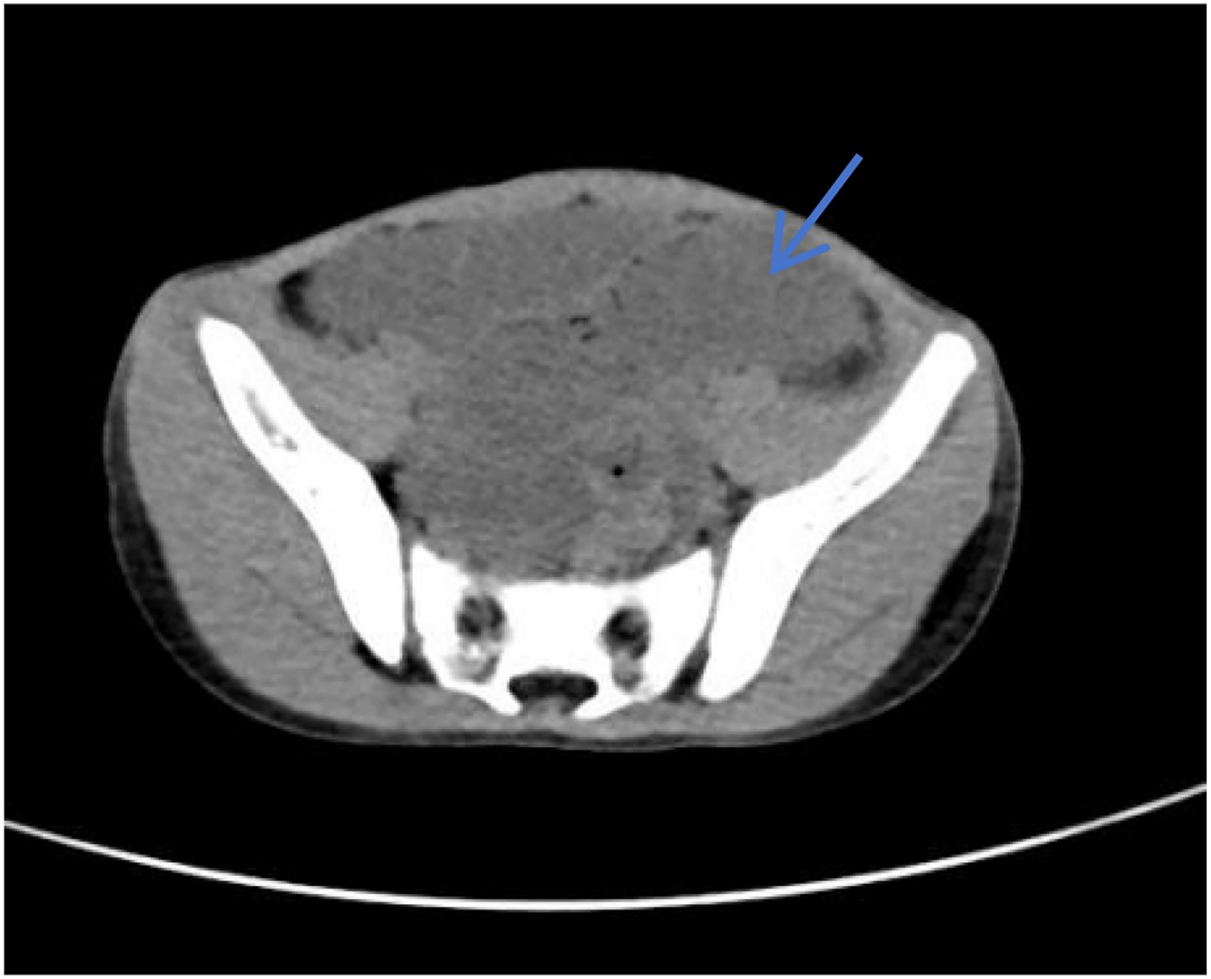
Large amount of ascites.

On admission, laboratory studies showed leukocytosis (18.48 × 10^9^/L); other laboratory findings were unremarkable. Notably, at the time of admission, the patient's abdominal pain had slightly improved compared to the onset, which posed a significant diagnostic challenge. CT imaging demonstrated internal hernia causing intestinal obstruction, complicated by peritonitis and massive ascites (Figures 1–3).

### 2.3 Surgical intervention

**Figure 4:**
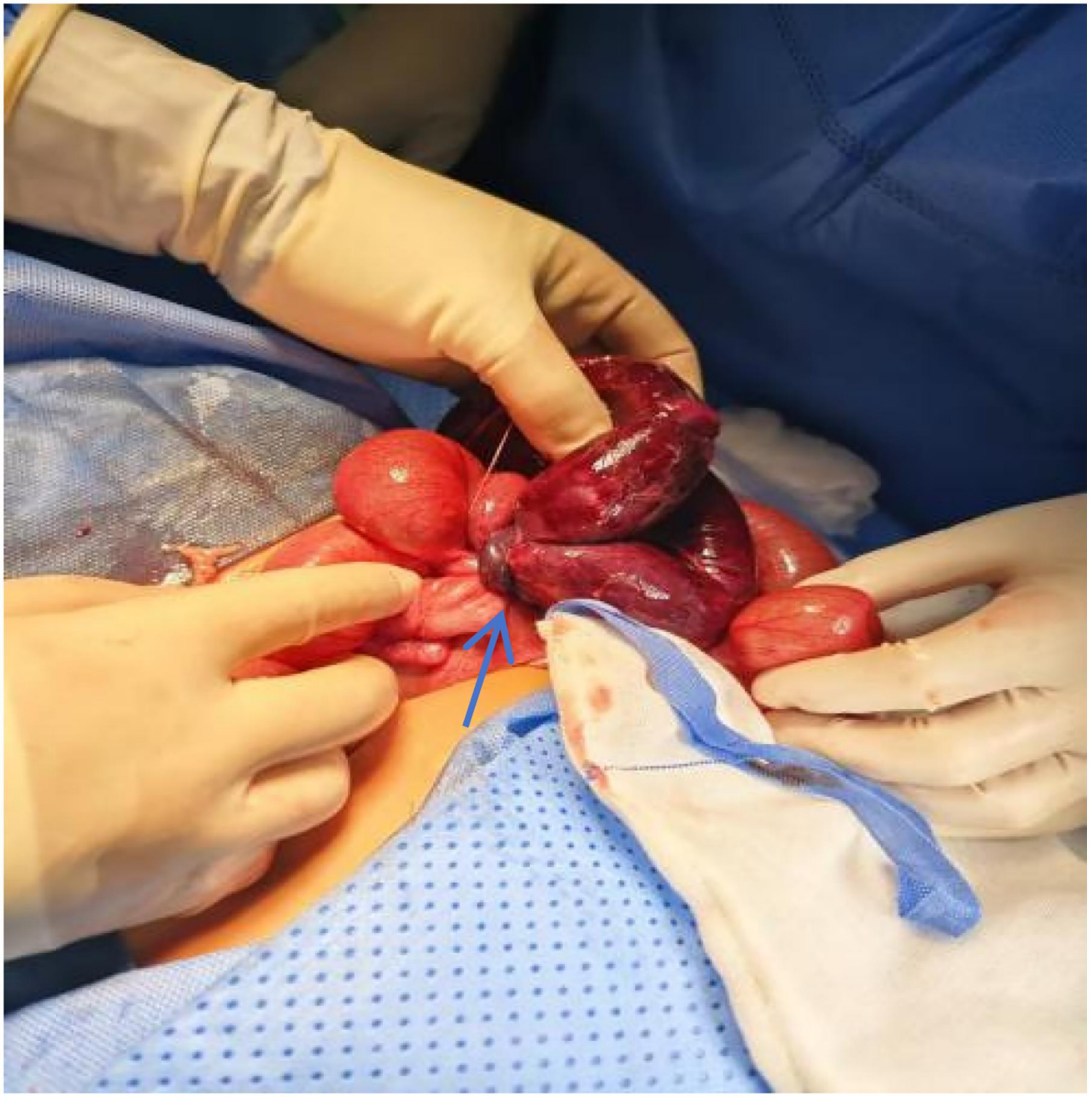
Proximal ileum herniated through the hernia ring, with ischemia and necrosis.

**Figure 5:**
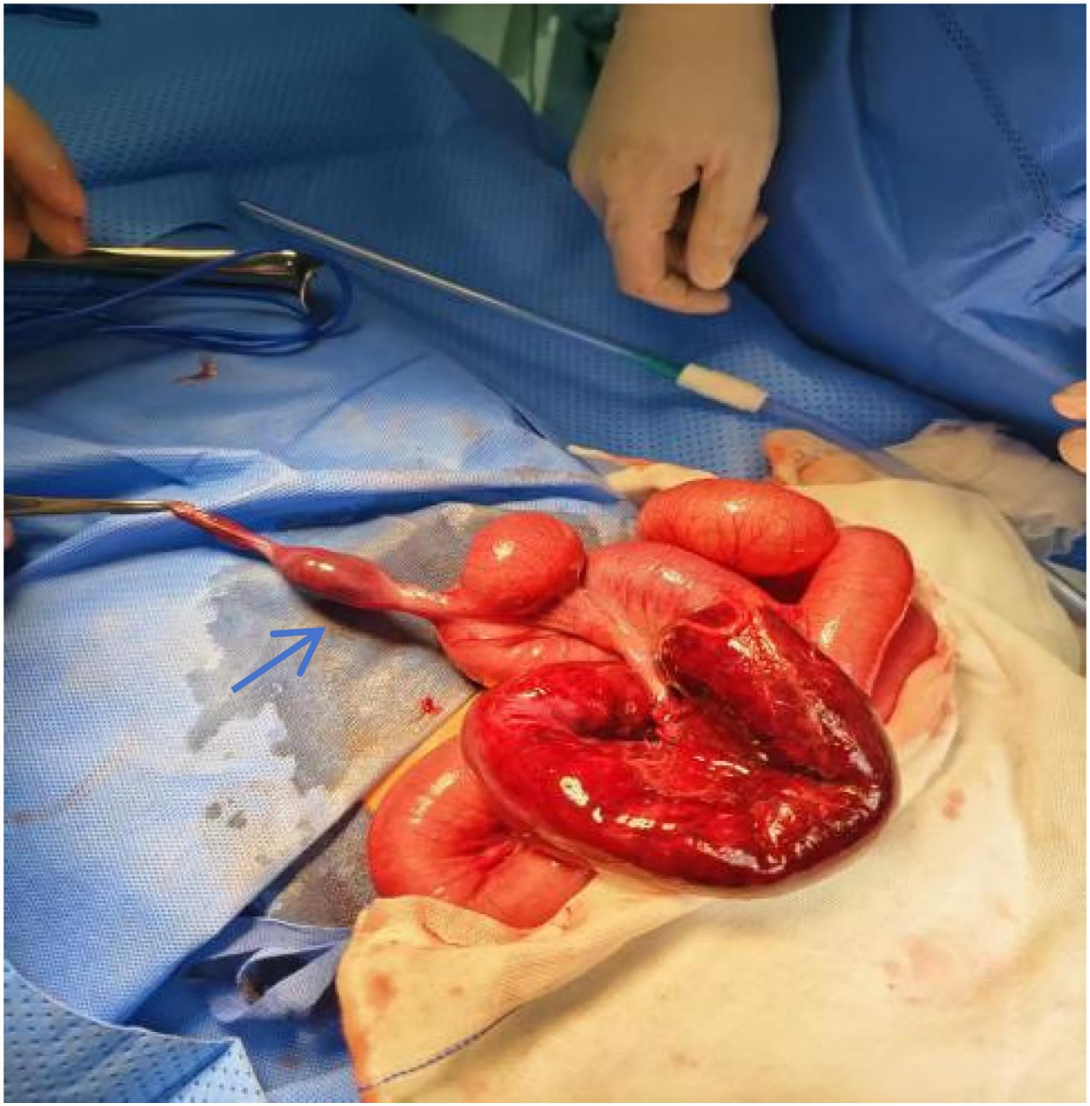
After division of the band adhesion, Meckel's diverticulum is visible.

Given the findings of high-grade bowel obstruction and suspected internal hernia, emergency surgery was indicated. Laparoscopic exploration followed by laparotomy confirmed the presence of a Meckel's diverticulum located approximately 40 cm proximal to the ileocecal valve. A distal diverticular band formed an adhesive ring, creating a hernia defect through which the proximal ileum had herniated (Figure 4), resulting in bowel compression and strangulated obstruction. Intraoperatively, the strangulated bowel segment was markedly dilated and appeared dark purple. After division of the band adhesion, decompression was attempted; however, bowel perfusion remained poor (Figure 5). Despite 30 min of warm compresses and pharmacologic intervention, no improvement was observed. Therefore, resection of the Meckel's diverticulum and 25 cm of necrotic ileum was performed, followed by end-to-end ileo-ileal anastomosis. Peritoneal drainage was placed, and the abdomen was closed in layers. The patient was followed up at 1 month and 6 months post-operatively at our outpatient clinic. During these visits, she showed satisfactory recovery. She reported no recurrent abdominal pain or symptoms suggestive of adhesive obstruction. Although she experienced occasional constipation, it was effectively managed with probiotics, and there were no hospital readmissions.

### 2.4 Treatment and histopathological confirmation

**Figure 6:**
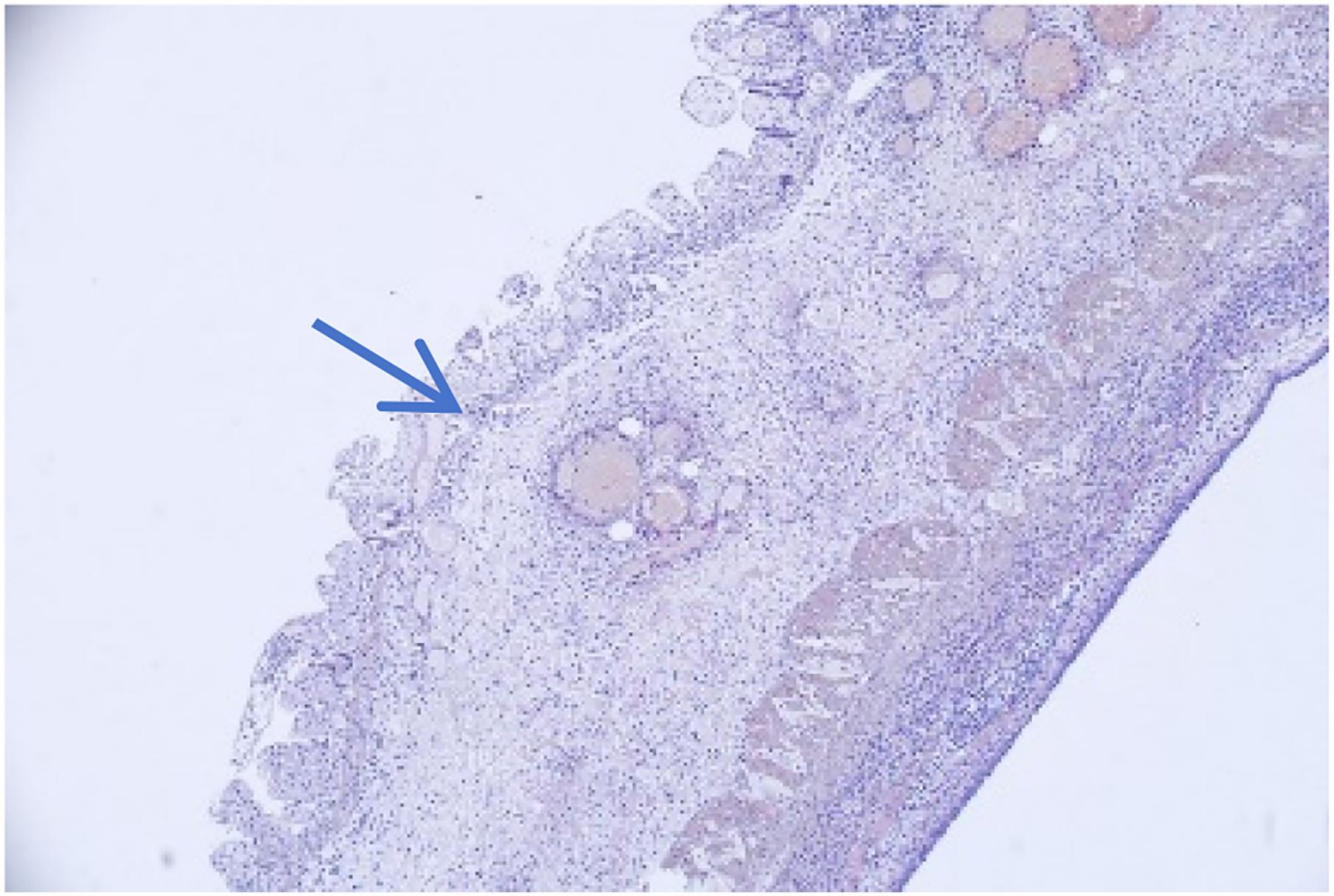
Histopathological appearance of the Meckel's diverticulum. (H&E staining,×100). The arrow indicates the site of localized ischemic necrosis.

**Figure 7:**
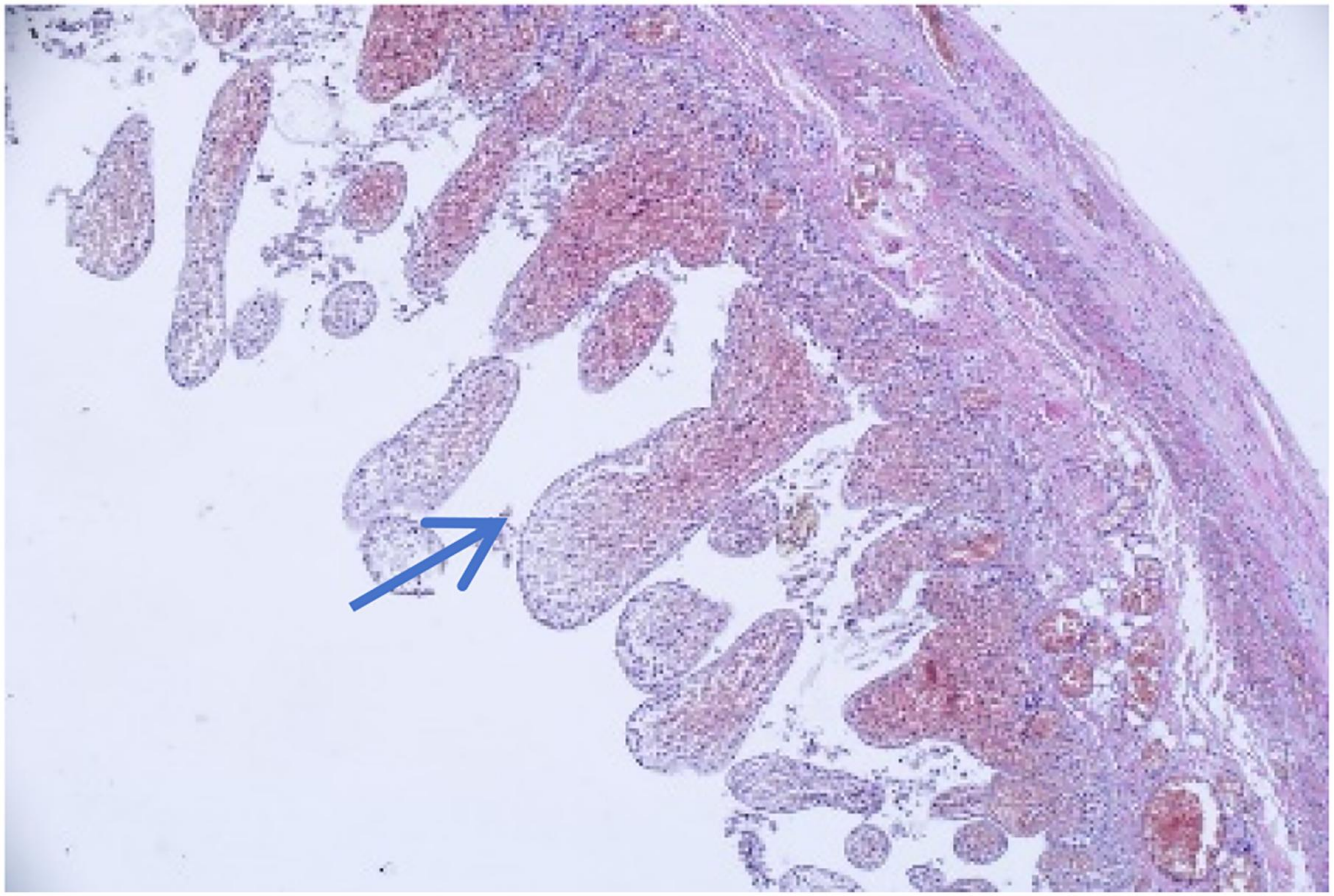
Histopathological appearance of the resected ileal segment. (H&E staining, ×100). The arrow indicates widespread ischemic necrosis of the bowel wall.

**Figure 8:**
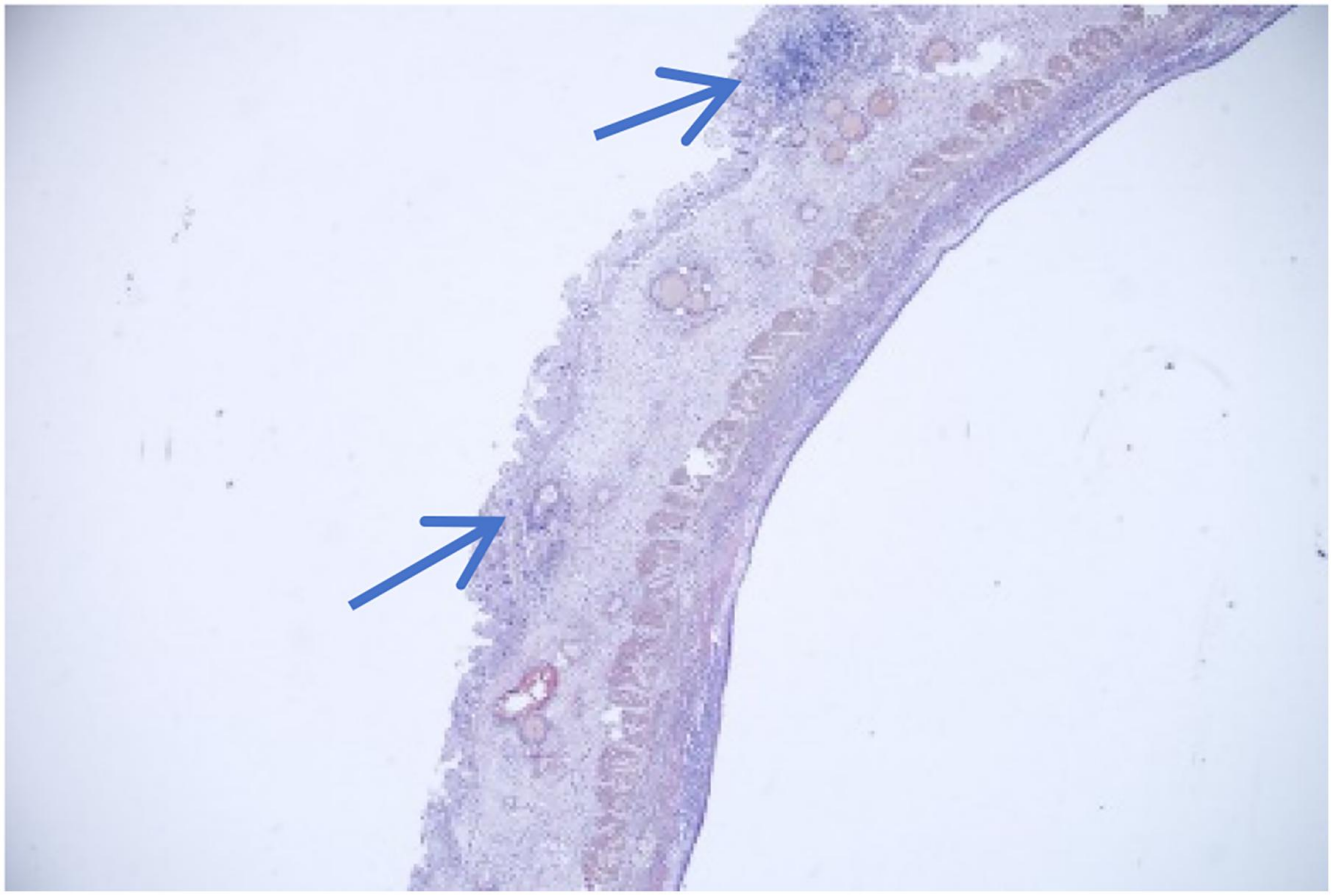
Histopathological appearance of the resected ileal segment. (H&E staining, ×40). The arrow highlights extensive inflammatory cell infiltration.

Histopathological examination of the resected specimen revealed a Meckel's diverticulum measuring 6 cm in length, with a basal diameter of 2 cm and a fibrous band diameter of 0.5–1.2 cm. No ectopic gastric or pancreatic tissue was identified. Analysis of the resected ileal segment demonstrated extensive transmural necrosis, significant hemorrhage, and massive inflammatory cell infiltration (Figure 6–8).

## 3 Discussion

The etiologies of intestinal obstruction in children are diverse and often present with nonspecific clinical manifestations. It is difficult to distinguish ordinary abdominal pain from severe pain caused by bowel strangulation and necrosis. In older children, increased pain tolerance may delay recognition of intestinal ischemia. Notably, the patient's abdominal pain slightly improved 20 h after onset; this may be attributed to the analgesic effect of the large volume of ascites or high pain tolerance, which represents a significant diagnostic trap in pediatric acute abdomen. Therefore, in pediatric patients without prior abdominal surgery or foreign body ingestion who present with unexplained acute abdomen, suspected obstruction, or strangulation, congenital anomalies must be strongly considered to avoid bowel necrosis, shock, or death due to internal hernia.

Meckel's diverticulum (MD) results from abnormal closure of the omphalomesenteric duct and is typically located on the antimesenteric border of the distal ileum (11). Only approximately 0.08% of the population develops clinical symptoms related to MD (12). Approximately 50% of symptomatic Meckel's diverticulum cases involve ectopic gastric mucosa, which can induce acid-mediated ulceration and perforation. For preoperative diagnosis of ectopic gastric or pancreatic tissue, Tc-99 m pertechnetate scanning is highly sensitive in children (13); however, this examination is not available in some primary care settings, which often contributes to low preoperative diagnostic rates and treatment delays.

Approximately 40% of symptomatic Meckel's diverticulum cases are complicated by intestinal obstruction (14, 15). While CT imaging is invaluable for identifying the pattern of obstruction and raising suspicion of an internal hernia, it is often challenging to achieve a definitive preoperative etiological diagnosis of a Meckel's diverticulum band (16). In our case, CT provided clear evidence of severe intestinal obstruction and internal hernia, which served as an urgent indication for exploratory surgery. Given the risk of rapid deterioration, we proceeded to emergency surgery to avoid delay, prioritizing the patient's clinical indications over definitive preoperative etiological confirmation.

## 4 Conclusion

The etiology of acute intestinal obstruction in children without prior abdominal surgery is complex. Internal hernia secondary to Meckel's diverticulum band adhesion, complicated by peritonitis and massive ascites, is rare but carries a high mortality risk. For children with acute intestinal obstruction and no history of abdominal surgery, congenital developmental anomalies should be considered a primary etiological factor. Although early CT diagnosis is valuable for localizing the obstruction and identifying the hernia, we must balance radiological findings with the patient's clinical progression. Surgical decision-making should prioritize clinical signs of systemic deterioration over radiological findings alone. Early surgical intervention, guided by both clinical suspicion and imaging, is critical to preventing bowel strangulation, necrosis, and shock, thereby significantly improving the patient's prognosis.

## Data Availability

The original contributions presented in the study are included in the article/Supplementary Material, further inquiries can be directed to the corresponding author.
